# Optimised power harvesting by controlling the pressure applied to molecular junctions†

**DOI:** 10.1039/d1sc00672j

**Published:** 2021-03-04

**Authors:** Xintai Wang, Ali Ismael, Ahmad Almutlg, Majed Alshammari, Alaa Al-Jobory, Abdullah Alshehab, Troy L. R. Bennett, Luke A. Wilkinson, Lesley F. Cohen, Nicholas J. Long, Benjamin J. Robinson, Colin Lambert

**Affiliations:** Physics Department, Lancaster University Lancaster LA1 4YB UK k.ismael@lancaster.ac.uk b.j.robinson@lancaster.ac.uk c.lambert@lancaster.ac.uk; The Blackett Laboratory, Imperial College London, South Kensington Campus London SW7 2AZ UK; Department of Physics, College of Education for Pure Science, Tikrit University Tikrit Iraq; Department of Physics, College of Science, University of Anbar Anbar Iraq; Department of Chemistry, Imperial College London, MSRH White City London W12 0BZ UK; Department of Chemistry, University of York Heslington York YO10 5DD UK

## Abstract

A major potential advantage of creating thermoelectric devices using self-assembled molecular layers is their mechanical flexibility. Previous reports have discussed the advantage of this flexibility from the perspective of facile skin attachment and the ability to avoid mechanical deformation. In this work, we demonstrate that the thermoelectric properties of such molecular devices can be controlled by taking advantage of their mechanical flexibility. The thermoelectric properties of self-assembled monolayers (SAMs) fabricated from thiol terminated molecules were measured with a modified AFM system, and the conformation of the SAMs was controlled by regulating the loading force between the organic thin film and the probe, which changes the tilt angle at the metal-molecule interface. We tracked the thermopower shift *vs.* the tilt angle of the SAM and showed that changes in both the electrical conductivity and Seebeck coefficient combine to optimize the power factor at a specific angle. This optimization of thermoelectric performance *via* applied pressure is confirmed through the use of theoretical calculations and is expected to be a general method for optimising the power factor of SAMs.

## Abstract 2

A major potential advantage of creating thermoelectric devices using self-assembled molecular layers is their mechanical flexibility.

## Graphical abstract

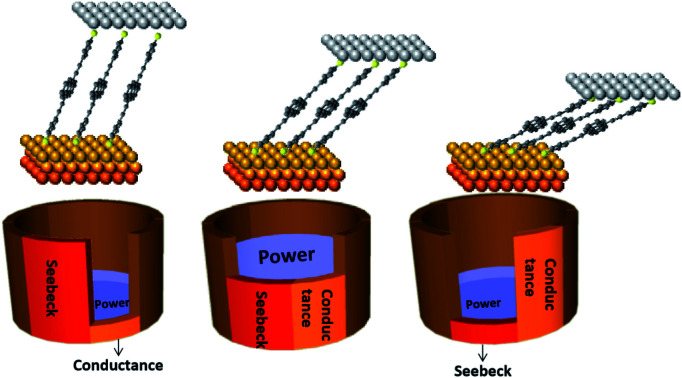

## Introduction

Thermoelectric devices which convert a temperature difference into electric power are attractive candidates in the recovery of waste heat.^2^ Molecular junctions are promising candidates for fabricating such devices, due to their low toxicity, high mechanic/synthetic flexibility and the ease through which they can be fabricated.^1,3–5^ More importantly, quantization of their electronic structure means that their thermopower can be enhanced when the Fermi level of the electrodes lies close to molecular frontier orbitals.^3,4,6–11^ Additionally, as highlighted in recent reviews^12,13^ Seebeck coefficients can also be tuned by varying the conformation and orientation of molecules suspended between two electrodes.^14–20^

A pre-requisite for controlling molecular conformation is to build a molecular junction with both a stable and a well-defined structure. Since single molecules are sensitive to their environment and to atomic-scale variations in the electrodes,^21,22^ self-assembled monolayer(s) (SAMs) are potentially a better starting point. Unlike single molecules, the configuration of molecules in SAMs is fixed due to intermolecular interactions, which can often result in crystalline or semi-crystalline structures.^23–26^ Previous literature has reported that the tilt angle of SAMs can be controlled by varying the loading force between the sample and probe using an AFM setup.^27–29^ In this work, we used a thermoelectric AFM system to characterize the thermoelectric properties of two different SAMs held a series of different tilt angles.

The SAM substrate and the metal-coated AFM probe are used as the source and drain in the fabrication of a standard ‘bottom up’ molecular junction system.^30–32^ To control the configuration of the molecules within such a sandwich, the loading force of the probe is varied with precise feedback control, using a laser deflection feedback loop.

**Fig. 1:**
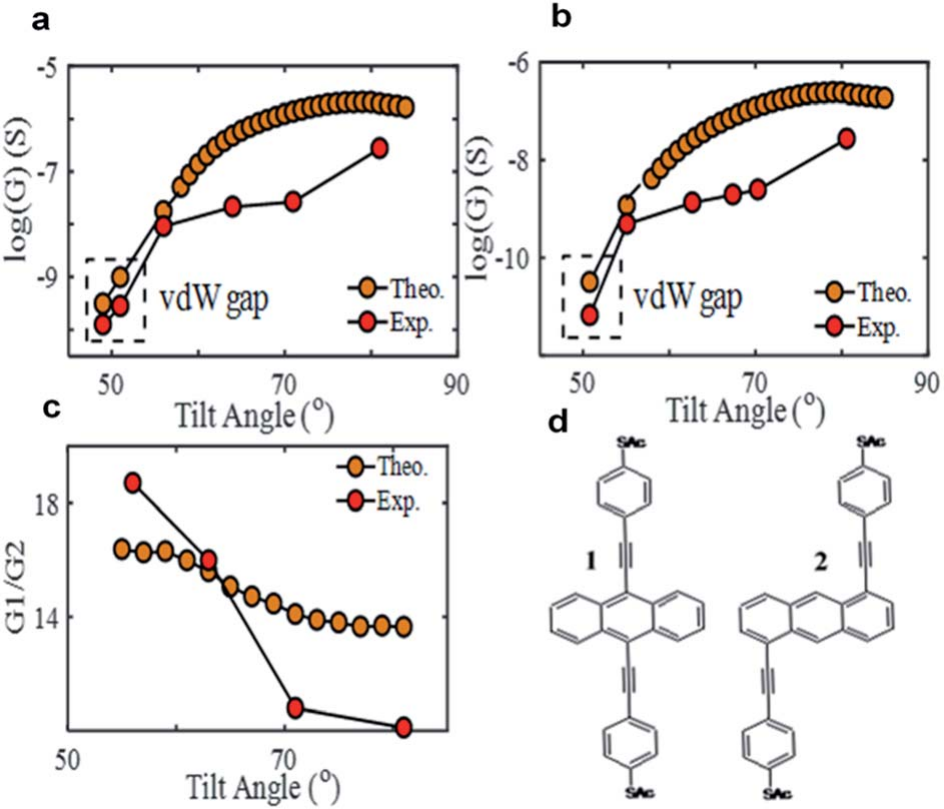
Electrical conductance of SAMs of **1** (a) and **2** (b) at different tilt angles, including a comparison between theory and experiment. Conductance ratio between SAMs of **1** and **2** at different tilt angle (c). Molecular structures of studied molecules^33,51^ (d).

**Scheme 1:**
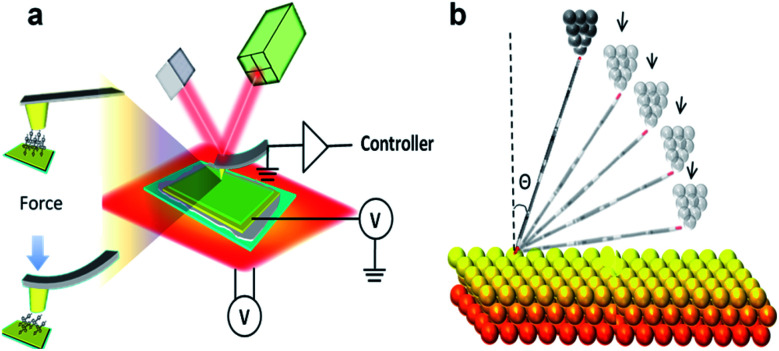
(a) Schematic illustration of molecular binding geome-try controlled by AFM, (b) scheme of pressure model.

We used this to study two molecular wires containing anthracene cores, linked to external electrodes through thiophenylalkyne termini (*via* two different connectivity's around the central anthracene unit). The synthesis of these wires, whose structures are shown in Fig. 1d, previously been reported,^33,34^ and we have shown that these are both highly-conductive and highly rigid, owing to the high-levels of conjugation and low conformational freedom presented by the alkynes used to bridge the anthracene core and the phenyl termini. This high rigidity means that when a loading force is applied the molecules tend to change their tilt angle, with respect to the substrate, rather than bend. The stiffness of the molecular thin film is estimated by AFM in PeakForce QNM mode. The measured Young's modulus for SAMs **1** is 2.1 GPa and for SAMs **2** is 2 GPa as shown in Fig. S2† (for more detail see Young's Modulus in the ESI†). This value (2 GPa) is about 1 order of magnitude higher than reported soft organic thin films such as synthetic glycosphingolipid^35^ or octanethiol^36^ based molecular layers, and comparable with other reported polyconjugation SAMs for example quarterthiophene.^37^ Due to the high rigidity of SAMs **1** and **2**, the bottom effect from the gold substrate was not considered a significant effect in this work (Scheme 1).

## Result and discussion

### SAM fabrication and identification

The SAMs were prepared by a standard procedure^23,38,39^ on template stripped (TS) gold,^40^ with detailed growing condition described in experiment section. SAM growth was monitored by co-growing a sample on a quartz crystal microbalance (QCM), and characterized by atomic force microscopy after growth. The quality of the SAMs was characterized by AFM topography. For both SAMs, the measured roughness is in the range of 0.1 to 0.2 nm, which is comparable with the roughness of a clean TS gold, and indicates a uniform molecular film on the substrate. The thickness of the molecular film is characterized by nanoscratching^41–43^ and measured to be about 1.2 ± 0.2 nm for molecule **1** and 1.2 ± 0.1 nm for molecule **2** (Fig. S1†). These values are comparable to the reported thickness of the SAMs^44^ of the same composition. The detailed thickness information of the nanoscratching measurements is listed in the Table S1.† Since density functional theory (DFT) calculations show that the lengths of both molecule **1** and **2** are about 1.9 nm, the tilt angle *Θ*, without any external pressure, is 57–61° for molecule **1** and 55–63° for molecule **2**. This tilt angle increases as the tip loading force increases. The change in *Θ* and the contact area with tip loading force is estimated *via* the Johnson–Kendall–Roberts (JKR) contact model (explained in SI).^29,45–47^ The nanomechanical parameters used in this model are obtained from peak force (PF) mode AFM with moderate frequency (2 kHz).^37,47,48^ The co-grown QCM substrate result suggests that the single molecular occupation area for molecule **1** is about 34 Å^2^, and for molecule **2** is about 38 Å^2^ (see the QCM work in the ESI†). These values are similar to our previously published data on the same SAMs^44^ (38 Å^2^ for molecule **1** and 39 Å^2^ for molecule **2**). Furthermore, they are comparable to the reported data on thiol anchored SAMs with similar oligo(phenyleneethynylene) back bone's, obtained from different methods, such as reductive desorption (40 Å^2^, OPE3 backbone)^41^ and high resolution XPS (28 Å^2^, OPE3 backbone).^16^

### Electric/thermoelectric characterization

Fig. 1 shows a comparison between the electrical conductivity of SAMs of **1** and **2**, measured by conductive AFM (cAFM) (details in experiment section) and predicted theoretically using DFT combined with quantum transport theory (for details see theory sections of the ESI†). Since the number of molecules contacting the probe increases with increasing loading force,^49,50^ we calibrated the measured conductance at different loading force to the single molecular scale, with the molecular occupation area estimated by QCM and probemolecule contact area estimated by a JKR model. The conductance distribution histograms and averaged IV curves of SAMs of **1** and **2** at different tilt angles are shown in the ESI,† and each point is averaged from at least 80 IV curves (Fig. S3–S6†).

A clear enhancement in electrical conductivity is observed as the tilt angle increases. The experimental measurements were made at four different tilt angles for **1** and five for **2** (excluding vdW gap), and compared with DFT simulations over a range of tilt angles (see Fig. S16 and S17 in the ESI†). The latter reveals a gradual enhancement in electrical conductance with increasing tilt angle, which is in excellent agreement with the measurements shown in Fig. 1a and b.

Magic ratio theory exists an intuitive way to predict the conductance ratio of molecular junctions with different connectivities to a large conjugated π system.^52,53^ This theory predicts that the conductance ratio 
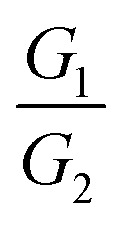
 of SAMs **1** and **2**, should be approximately 16, in agreement with recent experiments.^44^Fig. 1c is a plot of experimentally measured and theoretically predicted values of 
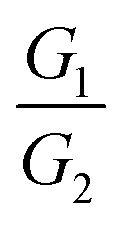
 at different tilt angles. Theory predicts that the conductance ratio is ∼16 when SAMs are in their natural form (tilt angle, *Θ* ≈ 55°), and decreases slightly (to ∼14) as the tilt angle is increased (brown-circles). The experimental results exhibit a similar decreasing trend in this ratio *vs.* the tilt angle, but with a larger decrease in the intensity (18.5–10, red-circles). This reduction in the conductance ratio is due to enhancement of intermolecular interactions that arise because of the larger loading force applied by the tip, which acts to quench the conductance ratio between the two SAMs.

The Seebeck coefficient of the SAMs were measured using a thermoelectric force microscopy (ThEFM) system, with a detailed explanation included in the ESI.† The histogram distribution and linear fit of thermal voltage *vs.* temperature difference at different tilt angle is also shown in the ESI (Fig. S7 and S8†).

**Fig. 2:**
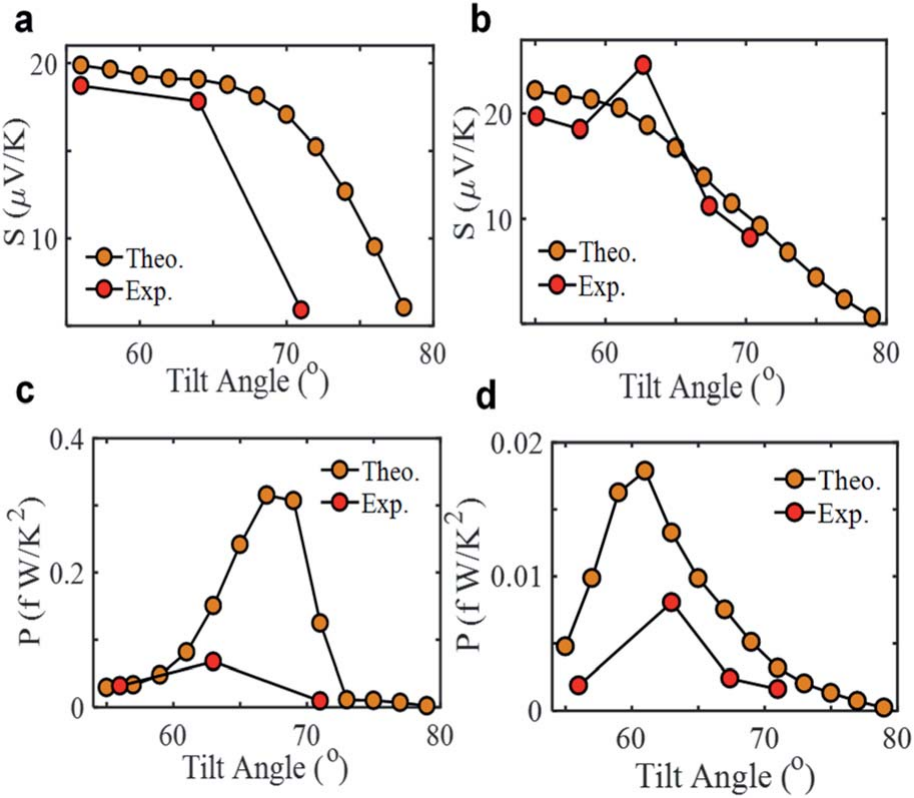
Seebeck coefficient of SAMs of **1** (a) and **2** (b). As well as, the experimentally measured and theoretical predicted power factor of SAMs of **1** (c) and **2** (d) at different tilt angles.

Fig. 2a and b show a clear decrease in the Seebeck coefficient as the tilt angle increases for SAMs of **1** and **2**. The DFT calculations exhibit a smooth reduction of the Seebeck value and an increase in conductance with increasing *Θ* for both SAMs, which agrees with the measured experimental trends.

The power factor of the molecular junction, *P* = *GS*^2^, is calculated for SAMs of **1** and **2** at different tilt angles both experimentally and theoretically (see Fig. 2c and d). At low tilt angles (SAMs in their native form, with a tilt angle of *Θ* ≈ 55°), the power factor is limited by the electrical conductance of the junction, *G*, whereas at high tilt angles (where the SAMs are compressed by the probe), the power factor is limited by the Seebeck coefficient, *S*. At intermediate the tilt angles, (*Θ* ≈ 65°) the power factor is optimized. Fig. S16 and S17† show that as the angle increases from approximately 55° to 80°, the transmission coefficient at the Fermi energy (and hence the conductance *G*) increases, but the slope at the Fermi energy (and hence the Seebeck coefficient *S*) decreases. Since the power factor is a product of *G* and *S*^2^, there is a competition between these two opposing trends and an optimum angle at which the product is maximised. The crucial point is that pressure can be used to tune the power factor, which we expect to be a generic property of SAMs. The precise value of the optimum angle, will of course depend on the chemical makeup of the monolayer and can only be obtained through a detailed DFT simulation.

**Fig. 3:**
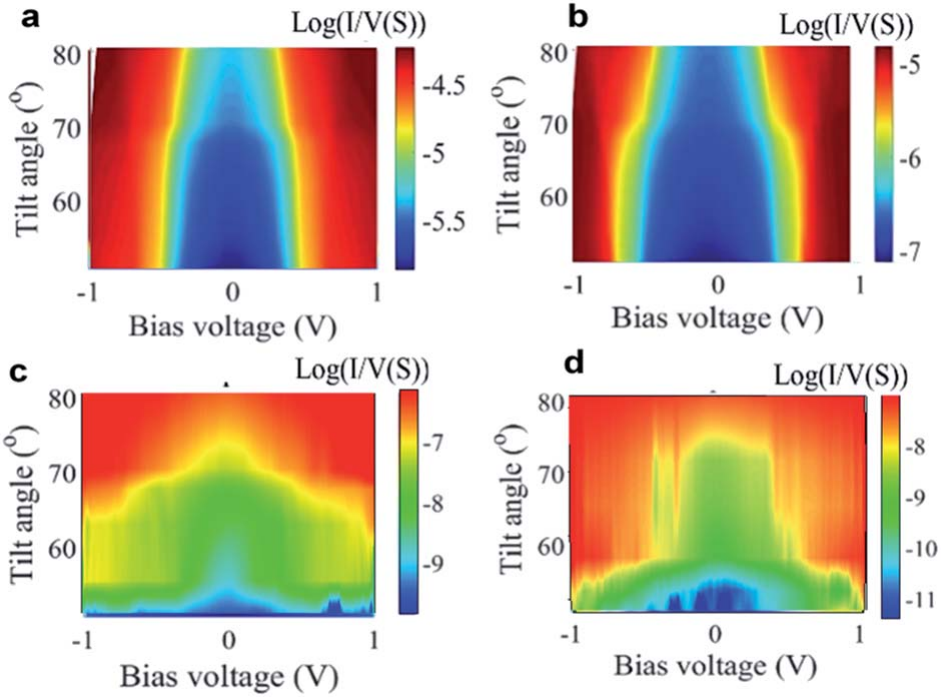
Mechanical gating of charge transport in molecular junctions. Two-dimensional visualization of *I*/*V* plotted *versus* bias voltage for SAMs of **1** and **2**. The top panels (a and b) are DFT calculations, while the lower panels (c and d) experimental results.

Fig. 3 shows that charge transport at finite biasses through SAMs is also sensitive to the tilt angle. Increasing the applied pressure leads to a higher conductance as shown in Fig. 1 and this behaviour is present at finite biasses in Fig. 3 both experimentally and theoretically (for more detail see Fig. S20–S23 in the ESI†).

In summary, we have demonstrated that both the Seebeck coefficient and electrical conductivity of SAM-based thermoelectric junctions can be effectively tuned through variation of an external applied pressure on two different molecular wires. Furthermore, we show that the power factor of these systems can be optimised through controlling the tilt-angle between a monolayer and its underlying substrate, with the application of ‘intermediate’ levels of pressure demonstrating the highest power factors. This work not only increases our understanding of how thermal voltages can be conducted through ultra-thin film materials, but also opens the way towards new methods of optimising the thermoelectrical performance of organic devices through controlling externally altering the conformation of their self-assembled mono layers. We are currently examining the tilt-angle dependence of electrical conductivity and Seebeck coefficient of SAMs formed from molecules with different structures to probe whether altering the molecule–substrate interface can achieve higher power factors.

## Author contributions

C. J. L., B. J. R. and A. K. I conceived the research. A. A., A. A., M. A. and A. A. carried out the calculations. X. W. and B. R. performed the measurements. N. J. L, T. L. R. B. and L. A. W. synthesised the molecules. All co-authors assisted in writing the manuscript. A. K. I, X. W, B. J. R. and C. J. L supervised the research and provided essential contributions to interpreting the results and drafting the manuscript.

## Conflicts of interest

There are no conflicts to declare.

## Supplementary Material

SC-012-D1SC00672J-s001
