# Supplementary material for: Optimised power harvesting by controlling the pressure applied to molecular junctions
Source: Chem Sci. 2021 Mar 4;12(14):5230–5. doi: 10.1039/d1sc00672j (PMC8179551; doi:10.1039/d1sc00672j)
Supplement: SC-012-D1SC00672J-s001 [file SC-012-D1SC00672J-s001.pdf]

Electronic Supplementary Material (ESI) for Chemical Science.
This journal is © The Royal Society of Chemistry 2021

Supporting Information

Optimized Power Harvesting by Controlling Pressure Applied to Molecular Junctions.

Xintai Wang,[a,b] Ali Ismael, [a,c,*] Ahmad Almutlg,[a] Majed Alshammari,[a] Alaa Al-Jobory, [a,d] Abdullah Alshehab,[a] Troy L. R. Bennett,[e] Luke A. Wilkinson,[e,f] Lesley F. Cohen, [b] Nicholas J. Long,[e] Benjamin J. Robinson[a,*] and Colin Lambert[a,*]

[a]Physics Department, Lancaster University, Lancaster, LA1 4YB, UK.

[b]The Blackett Laboratory, Imperial College London, South Kensington Campus, London, SW7 2AZ, UK.

[c]Department of Physics, College of Education for Pure Science, Tikrit University, Tikrit, Iraq.

[d]Department of Physics, College of Science, University of Anbar, Anbar, Iraq.

[e]Department of Chemistry, Imperial College London, MSRH, White City, London, W12 0BZ, UK.

[f]Department of Chemistry, University of York, Heslington, York, YO10 5DD, UK.

*To whom correspondence should be addressed. e-mail: k.ismael@lancaster.ac.uk; b.j.robinson@lancaster.ac.uk; c.lambert@lancaster.ac.uk;

Table of Contents:

Section 1: Experimental details.

Section 2: Theoretical details.

Reference

Experimental Section

1.1 Au preparation

The ultra-flat gold was prepared by modifying the template stripped (TS) method of Whitesides and Pinkhassik [1] [2] on Silicon. The Si wafer (in 5 mm x 5 mm) was ultra-sonicated in acetone, methanol and isopropanol in succession and then cleaned with oxygen plasma for 5 minutes. The cleaned wafer was glued onto the gold deposited Si substrate with Epotek 353nd epoxy adhesive to form Si/Glue/Au/Si sandwich structure. After 40 minutes curing of glue at 150 ^{o}C, the ultra-flat TS gold was obtained by eliminating the Si contact with Au without adhesive by a knife. The prepared gold was scanned by AFM for 3-5 random spots for quality control. For all cases, the average roughness of the gold was less than 0.2 nm.

1.2 SAMs growth

A 1 mM solution of each molecule was prepared by dissolved in toluene, with 10 minute deoxygenation by nitrogen bubbling. The freshly cleaved Au^{TS} without any treatment was immersed into the solution, and incubated for 24 hours in vacuum.

After SAM growth, the sample was rinsed with toluene, ethanol and isopropanol several times to remove physisorped molecules. After rinsing the sample was blown with nitrogen for drying, and incubated in vacuum oven (10^{-2} mbar) overnight at 35 ^{o}C for solvent evaporation.

1.3 SAMs characterization

QCM measurement:

QCM was used to quantify the amount of adsorbed molecule on Au surface.

A new gold QCM crystal (5mm diameter, f_0 = 10 MHz, from icryst) was cleaned by oxygen plasma for 10 minutes, immersed in hot DMF (100 ^{o}C) for 2 hours, and in room temperature DMF overnight, washed with ethanol and isopropanol, and dried in vacuum oven for 20 hours at 35 ^{o}C. The cleaned QCM substrate was used for SAMs growth, the growing condition was the same as SAMs growing on AuTS.

The QCM measurement was operated by an openQCM system. The resonance frequency of the substrate before and after SAMs growth was recorded, and the frequency difference, Δf, implied the amount of molecules adsorbed an substrate surface. The relationship can be expressed by the Sauerbrey equation[3]:

$$n = \frac{-\Delta f \times A \times k \times N_A}{M_w}$$

$$k = \frac{\sqrt{\mu * \rho}}{2 * f_0^2}$$

Where n is the amount of molecule adsorbed on Au surface, A is the electrode area, N_A is the Avogadro's number, M_w is the molecular weight, μ is the shear modulus of quartz, ρ is the density of quartz, and f_0 is the initial frequency.

AFM and nano-scratching [4, 5] analysis:

The SAM sample on Au^{TS} was measured by AFM (multi-mode 8, Brucker) in peak force mode. The roughness of the sample surface was obtained through the use of nano-scope 9.0 software. For both SAMs, the measured roughness was comparable with a freshly cleaved Au^{TS}, which indicates a uniform molecular layer on the substrate surface.

The nano-scratching was performed in contact mode at high set force (F = 15 - 40 nN) using a soft probe (Multi-75-G, k = 3 N/m) to 'sweep away' the molecular film from a defined area. The topography of sample after scratching was again characterized in peak force mode,

where the scratched window is easily observed. Nano-scratching was also conducted on a bare gold sample under the same conditions to ensure no gold is scratched away in used force range. The height difference between the scratched part and un-scratched part indicates the thickness of SAMs.

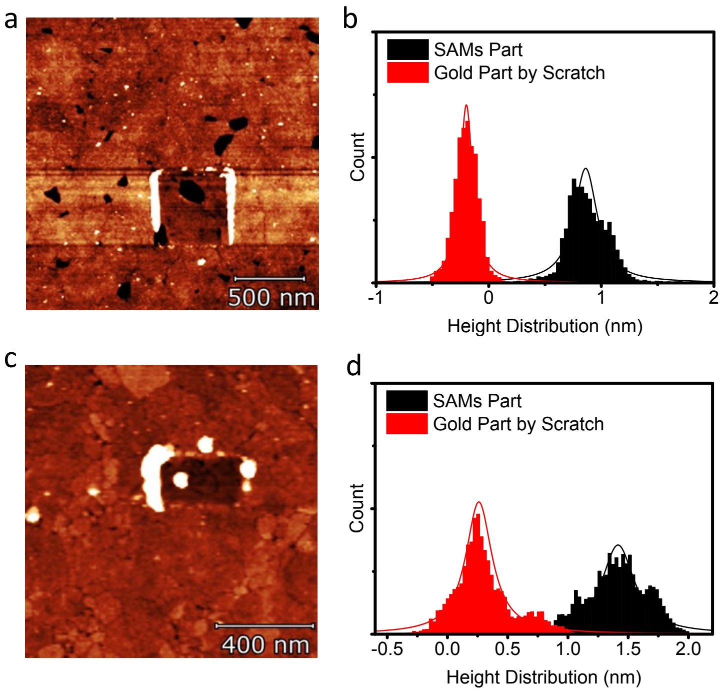

Figure S1. (a,c) the AFM topography of SAMs **1** and **2**, the middle square was the Nano-scratched part. (b,d) Height distribution of SAMs **1** and **2** at scratched and un-scratched part.

Figure S1 (b,d) shows the height distribution of SAMs **1** and **2** in scratched and un-scratched parts with a Gaussian distribution. The difference in the peak of the height-distribution indicates the SAMs thickness.

The nano-scratching was done on 2 random spots on each sample, and the result was used to compare with our previous result done on same SAMs system with same experimental method. The obtained result was similar, as listed in Table S1.

Table S1. Film thickness of SAMs 1 and 2 by Nano scratching, from this work and our previous work.[6]

SAMs	Sample#	Spots	Film Thickness (nm)	std (nm)	Ref.
**1**	1	1	1.2	0.2	This work
**1**	1	2	1.1	0.2	This work
**1**	2	1	1.1	0.2	6
**1**	2	2	1.3	0.2	6
**1**	3	Average from multiple spots	1.1	0.1	6
**2**	1	1	1.1	0.1	This work
**2**	1	2	1.2	0.1	This work
**2**	2	1	1.3	0.1	6
**2**	2	2	1.2	0.1	6
**2**	3	Average from multiple spots	1.2	0.1	6

The tilt angle of the molecule in SAMs form, ϴ, can be calculated through use of the following equation [7]:

$$\theta = 90^{o} - asin(\frac{Film\,Thickness}{Molecule\ Length})$$

1.4 Electrical characterization

The electrical conductivity of the film was characterized by a conductive AFM setup based on a Multi-mode 8 AFM instrument (Bruker Nano Surfaces). The bottom gold substrate was used as the source, and a Pt/Cr coated probe (Multi75 E, BugetSensors) was used as the drain. The force between probe and monolayer was controlled by the deflection error set point. The triangular shape AC bias was added between the source and drain by a voltage generator (Aglient 33500B), the source to drain current was acquired by a current pre-

amplifier (DLPCA200, Femto) providing current-to-voltage conversion. The I-V characteristics were obtained by Nanoscope 8 controller simultaneously collecting drive bias and current with subsequent correlation of these values at each time point.

1.5 Young's modulus estimation

Young's modulus was determined by an AFM setup (Multi-Mode 8) in Peak Force Quantitative Nanomechanical Nanoscale Mechanical Characterization (PF QNM) mode[8, 9]. The PF QNM mode AFM operated at 2 kHz frequency with 150 nm distance. Force spectroscopy was recorded based on the rapid collection of point by point force curves. Young's modulus was calculated from DMT model and averaged from all force curves by Nanoscope Analysis.

For each sample the peak-force map was operated on 3 random spots, and each spot with area of 1μm x 1μm. The map resolution was set to be 32 x 32 pixels, and 1 force curve was operated on each pixel. The spring constant of the tip was calibrated by thermal tune and the tip radius was characterized by SEM.

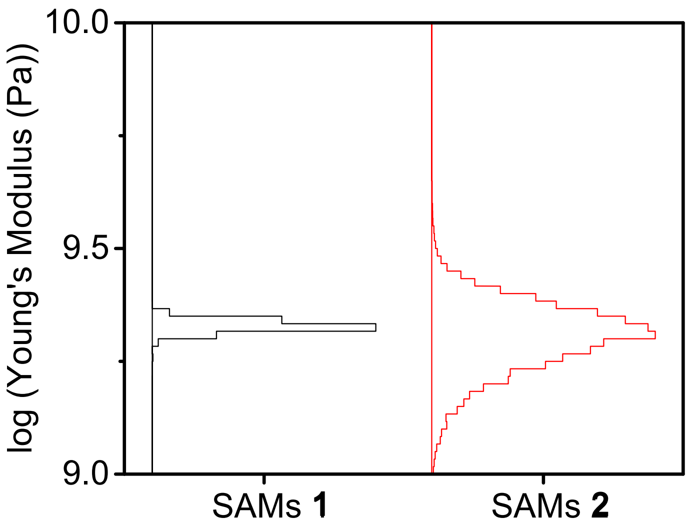

Figure S2. Young's Modulus distribution obtained by AFM in Peak Force QNM Mode for SAMs **1** and **2**.

1.6 Contact area estimation

The contact area between the probe and the sample was estimated via a JKR model, where the contact radius, r, is calculated from equation:

$$r = (F \times R \times \frac{1}{Y})^{\frac{1}{3}}$$

$$\frac{1}{Y} = \frac{3}{4} \times (\frac{1 - v_1^2}{E_1} + \frac{1 - v_2^2}{E_2})$$

Where r is the contact radius, F is the loading force from probe to sample, R is the radius of the probe, v1 and v2 are the Poisson ratio of the material and E1 and E2 are the Young's Modulus for probe and SAMs. The radius of the probe was obtained from SEM image, and estimated to be 25 nm. The Young's modulus was obtained from AFM in peakforce QNM mode with details mentioned in previous section, which was about 2 GPa for both SAMs. Other parameters were obtained from literature working on similar systems. The amount of molecules contact with probe was calculated from equation $= \frac{Contact\ Area}{Occupation\ Area\ per\ molecule}$, occupation area per molecule was estimated from QCM measurement mentioned in previous section.

1.7 Tilt angle estimation

The tilting angle under different loading forces was estimated through use of a JKR model [7, 10]:

$$\delta = \frac{r^2}{R} - \sqrt{\frac{8\pi\tau a}{3Y}}$$

$$\tau = \frac{2P}{3\pi R}$$

δ is the tip-substrate distance shortened due to the tip loading force. P is the tip-sample adhesion force obtained from peak force mode, other parameters as described in contact area estimation.

The tilt angle, Ɵ, was calculated from equation:

$$\theta = 90^o - \mathrm{asin}\left(\frac{df - \delta}{dm}\right)$$

Where df is the film thickness and dm is the molecular length.

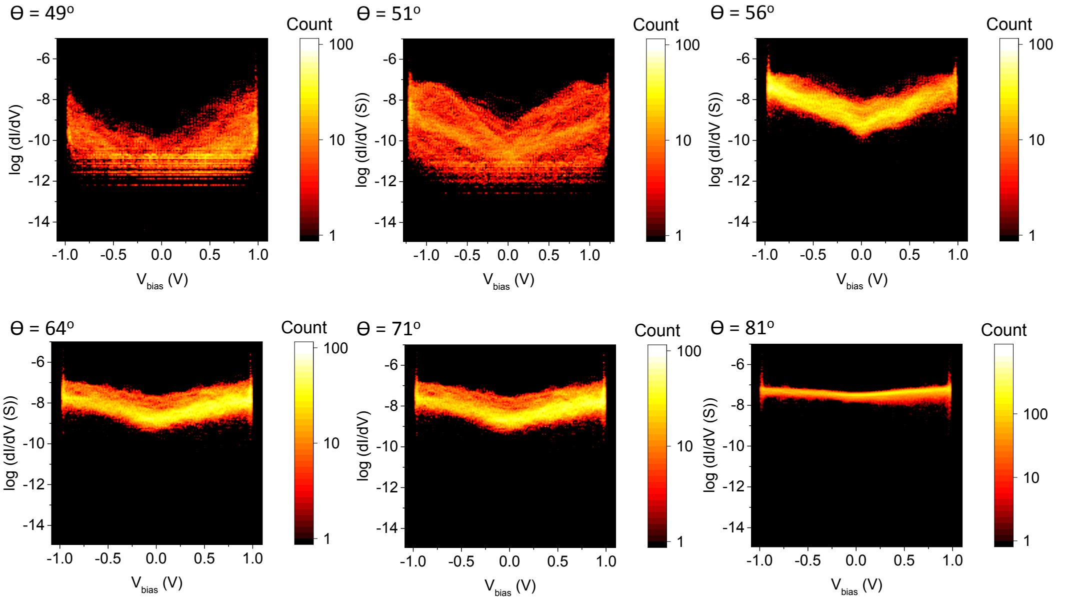

Figure S3. The statistical curves of molecular conductance, log (dI/dV (S)) vs. V_{bias} of SAMs **1** at different tilt angles.

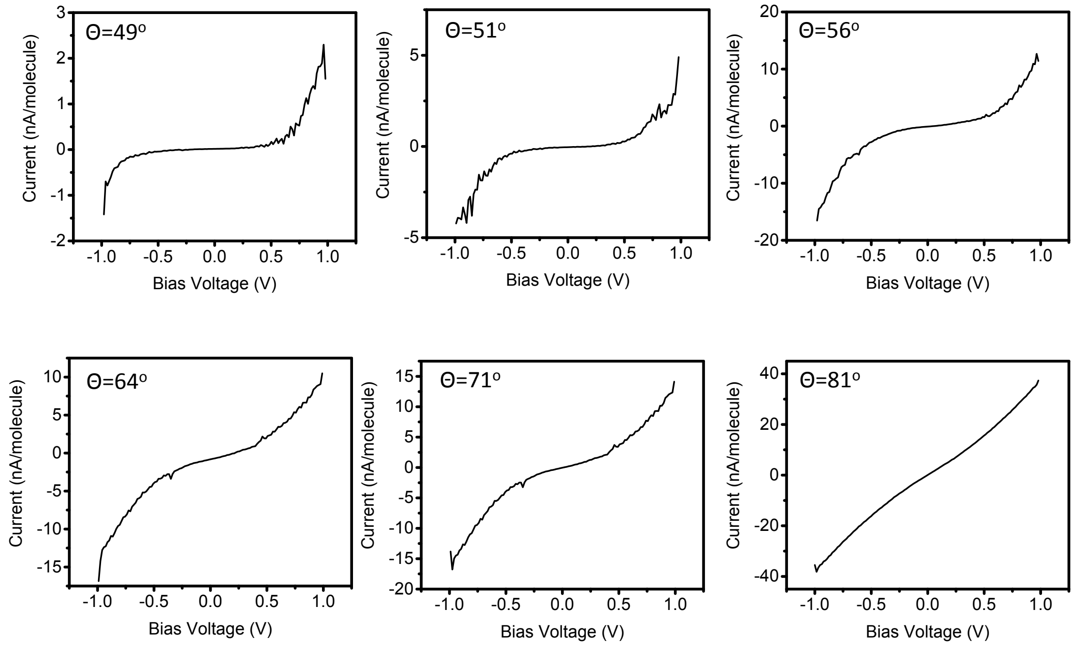

Figure S4. Averaged IV curve of SAMs **1** at different tilt angles. Approximately 80-200 I-V curves were averaged to obtain each I-V curve.

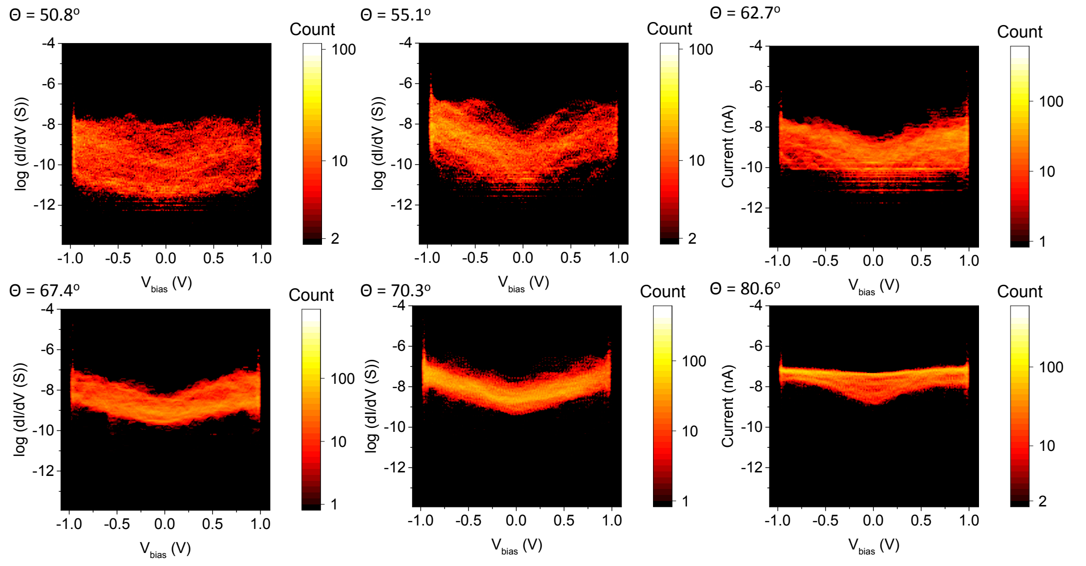

Figure S5. The statistical curves of molecular conductance, log (dI/dV (S)) vs. V_{bias} of SAMs **2** at different tilt angles.

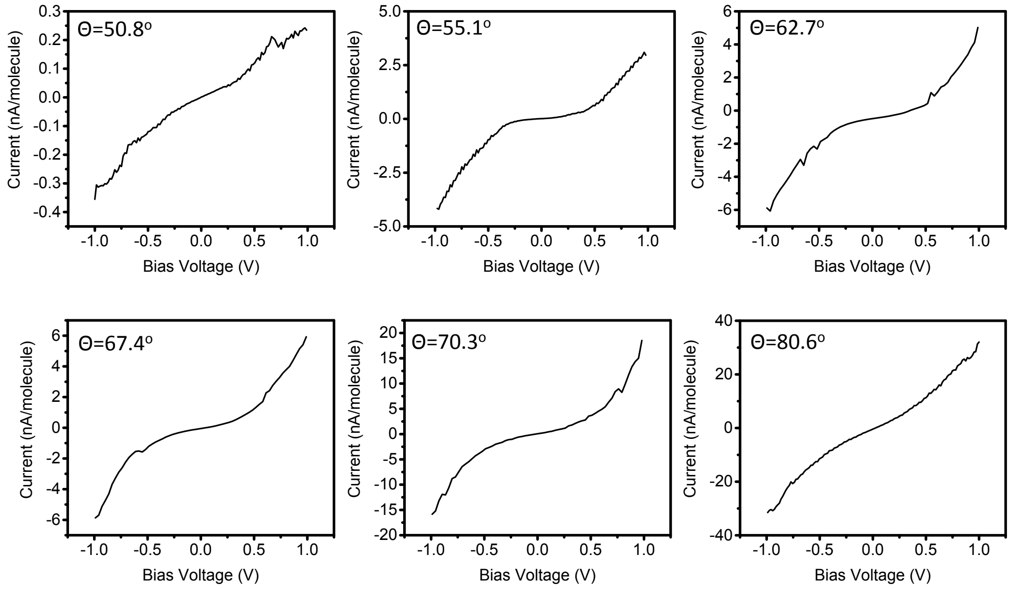

Figure S6. Averaged IV curve of SAMs **2** at different tilt angles. Approximately 80-200 I-V curves were averaged to obtain each I-V curve .

1.8 Seebeck Characterization

The Seebeck coefficient of SAMs were obtained through use of Thermal-Electrical Atomic Force Microscopy (THEFM), which is a modified version of the cAFM used for our electrical transport measurements. The probe was coated with 100 nm Au by thermal evaporation for voltage stabilization. A Peltier stage controlled by a voltage generator (Aglent 33500B with

broad-band amplifier) was used for substrate temperature control, and the temperature difference between sample and probe, ΔT, can be created. A Type T thermal couple was used to quantify this ΔT. The thermal voltage between sample and probe, ΔV_{Therm}, was amplified by a high impedance differential pre-amplifier (SR551, Standford Research System). The signal was passed through a low pass filter and recorded by the computer. The linear regression of ΔV_{Therm} vs. ΔT was plotted, and the slope of the linear curve was the Seebeck Coefficient of the system.

The tilting angle of SAMs was controlled by loading force between sample and probe as described in the previous section.

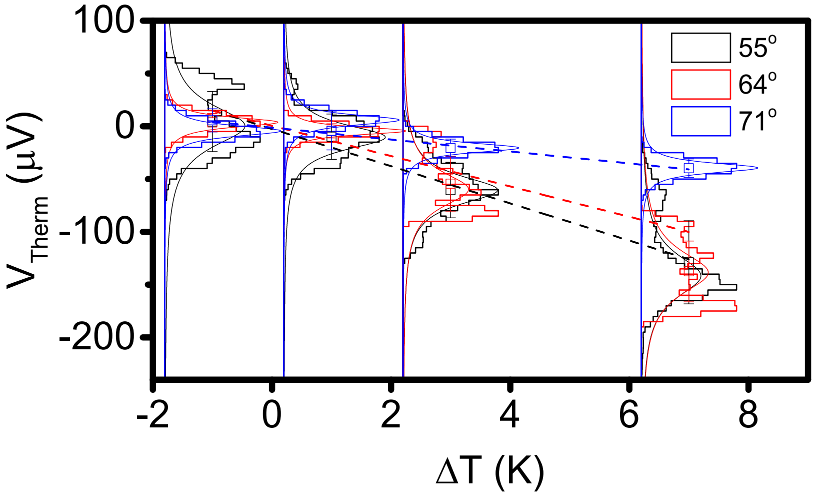

Figure S7. The thermal voltage distribution at different ΔT with different tilt angle for SAMs **1**

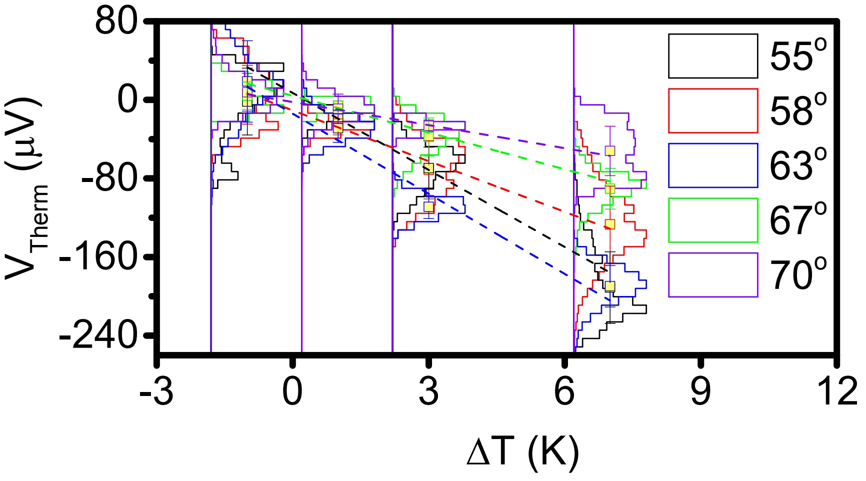

Figure S8. The thermal voltage distribution at different ΔT with different tilt angle for SAMs **2**

2. DFT and Transport Calculations

2.1 Optimised DFT Structures of Isolated Molecules

Using the density functional code SIESTA, the optimum geometries of the isolated molecules **1** and **2** were obtained by relaxing the molecules until all forces on the atoms were less than 0.01 eV / Å as shown in Figure S8.[11, 12] A double-zeta plus polarization orbital basis set, norm-conserving pseudopotentials, an energy cut-off of 250 Rydbergs defined the real space grid were used and the local density approximation (LDA) was chosen to be the exchange correlation functional. We also computed results using GGA and found that the resulting transmission functions were comparable with those obtained using LDA.[13, 14]

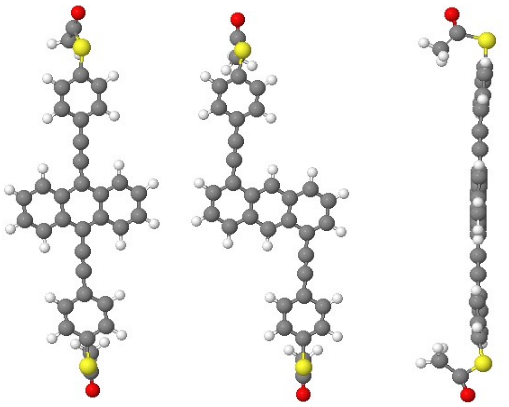

1 2 side-view

Figure S9. Fully relaxed isolated molecules **1** and **2**. Key: C = grey, H = white, O = red, S = yellow (synthesis reported previously)[15, 16]

2.2 Frontier orbitals of the molecules

The plots below show isosurfaces of the HOMO, LUMO, HOMO-1 and LUMO+1 of isolated molecules **1** and **2**.

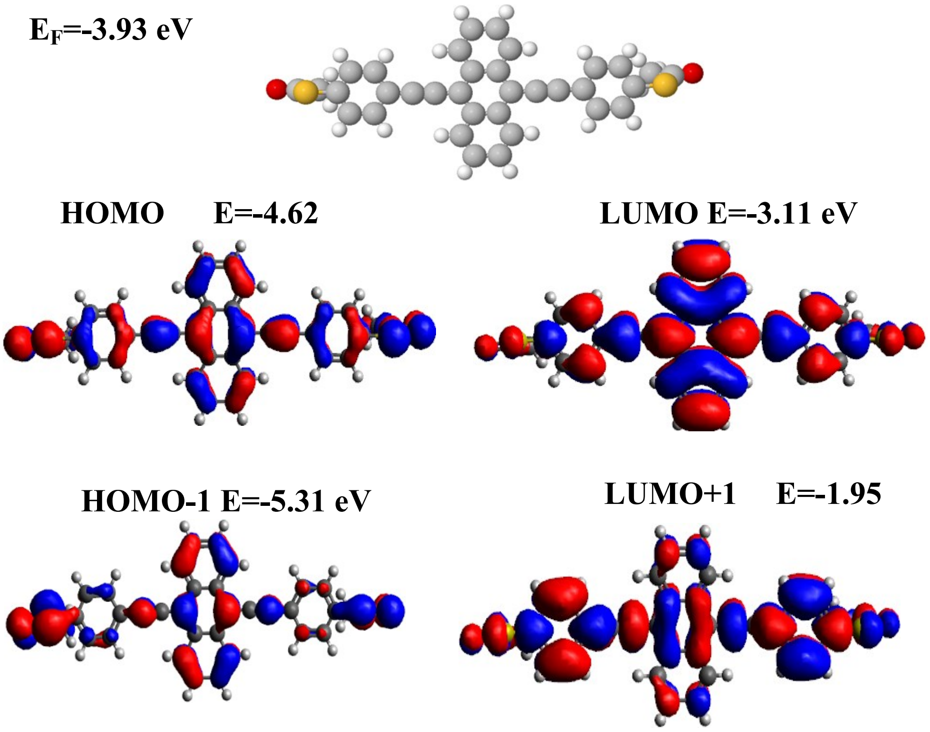

Figure S10. Wave function for **1**. Top panel: Fully optimised geometry of **1**. Lower panel: HOMO, LUMO, HOMO-1 and LUMO+1 along with their energies

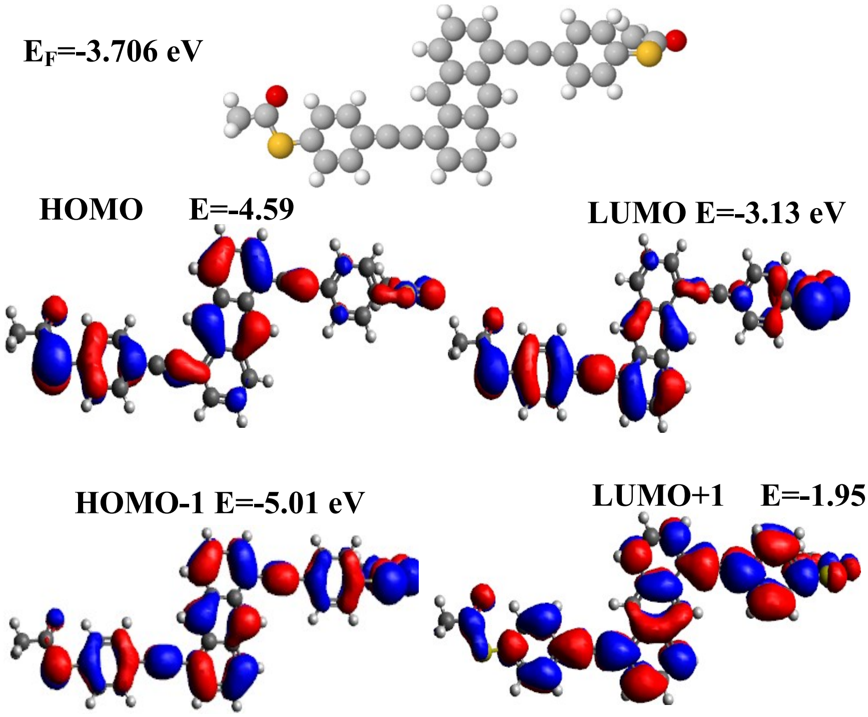

Figure S11: Wave function for **2**. Top panel: Fully optimised geometry of **2**. Lower panel: HOMO, LUMO, HOMO-1 and LUMO+1 along with their energies.

2.3 Binding energy of molecules on Au

To calculate the optimum binding distance between thiol anchor groups and Au(111) surfaces, we used DFT and the counterpoise method, which removes basis set superposition errors (BSSE). The binding distance d is defined as the distance between the gold surface and the S terminus of the thiol group. Here, compound 1 is defined as entity A and the gold electrode as entity B. The ground state energy of the total system is calculated using SIESTA and is denoted E_{AB}^{AB}. The energy of each entity is then calculated in a fixed basis, which is achieved using ghost atoms in SIESTA. Hence, the energy of the individual 1 in the presence of the fixed basis is defined as E_{A}^{AB} and for the gold as E_{B}^{AB}. The binding energy is then calculated using the following equation:

$$Binding\ Energy = E_{AB}^{AB} - E_{A}^{AB} - E_{B}^{AB} \qquad \text{(S1)}$$

We then considered the nature of the binding depending on the gold surface structure. We calculated the binding to a Au pyramid on a surface with the S atom binding at a 'top' site and then varied the binding distance d. Figure S11 (left) shows that a value of d = 2.4 Å gives the optimum distance, at approximately 0.8 eV. As expected, the thiol anchor group binds favorably to under-coordinated gold atoms.

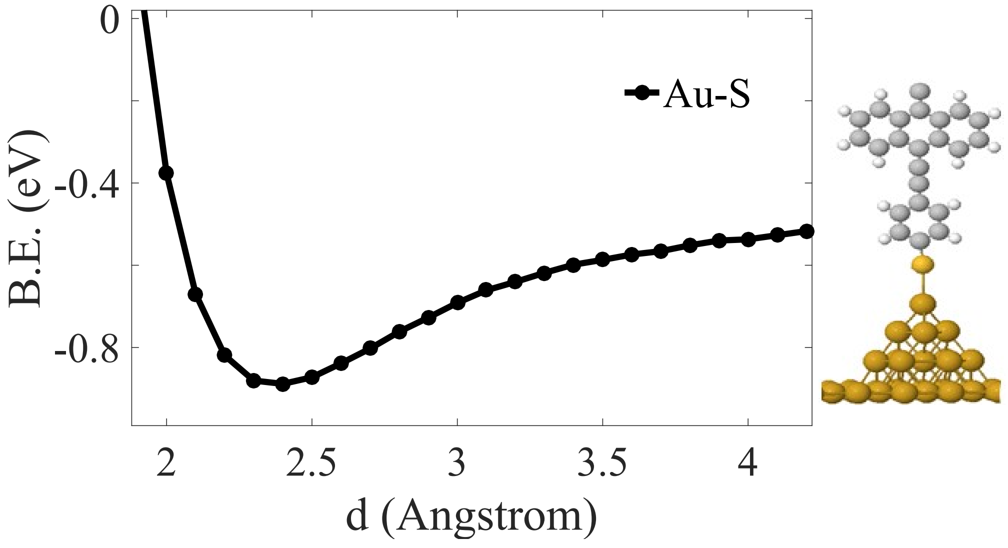

Figure S12. Example binding energy plot of **1**, for thiol anchor Au-S (left), with its idealised ad-atom configuration at the Au lead interface Au-S. Key: C = grey, H = white, S = light yellow, Au = dark yellow.

2.4 Optimised DFT Structures of Compounds in their Junctions

Using the optimised structures and geometries for the compounds obtained as described in section 2.1 (above), we again employed the SIESTA code to calculate self-consistent optimised geometries, ground state Hamiltonians and overlap matrix elements for each metal-molecule-metal junction. Leads were modelled as 625 atom slabs. The optimised structures were then used to compute the transmission curve for each compound. The DFT optimised geometries are shown here, in Figure S12. Note: there is a tilt angle range for each compound, which is presented in section 2.5.

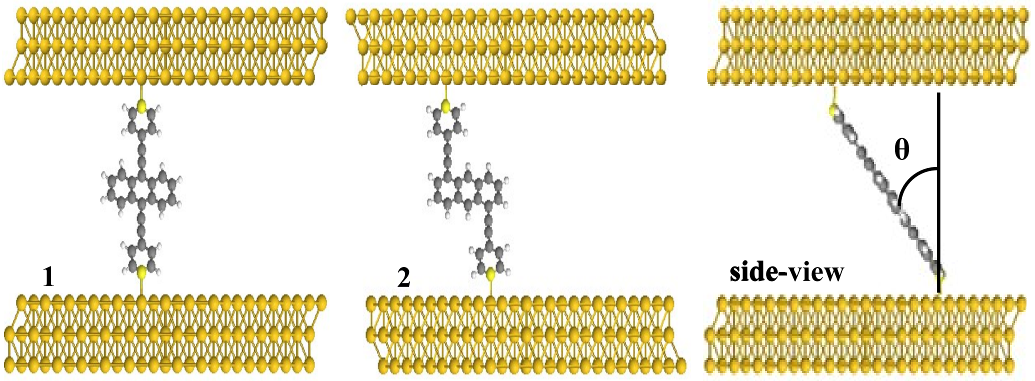

Figure S13. Optimised structures of **1** and **2**. Tilt angle (side-view)

2.5 The tilt angle (θ)

In this section, we determine the tilt angle, θ, of each compound on a gold substrate, which corresponds to the experimentally measured most-probable break-off distance. Table S2 shows a range of tilt angles calculated from the film thickness for each molecule. Break-off distance values suggest that compound-1 tilt with angle θ ranging from 57° to 61° and compound-2 55° to 63°, as shown in Figure S13.

Table S2: Experimental break-off distance and equivalent tilt angle (θ)

Compound	Experimental film thickness (nm)	Experimental film roughness (nm)	Equivalent experimental tilt angle (θ)	Equivalent theoretical tilt angle (θ)
**1**	1.17	0.43	57°-61°	57°-61°
**2**	1.15	0.09	55°-63°	55°-63°

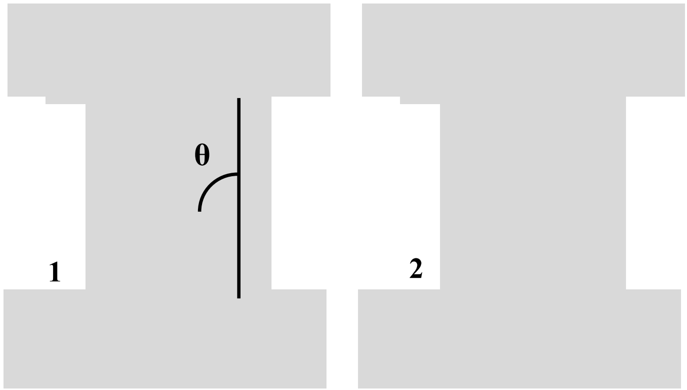

Figure S14. Optimised structures of **1** and **2.**

2.6 Beyond the optimised tilt angle (θ)

After finding the optimised tilt angle for molecule **1** and molecule **2,** these angles were varied by up to 80° degrees when measuring the conductance, and up to 70° degrees for Seebeck measurements. The difference between *G* and *S* tilt angles arose from the need to heat up the tip during the Seebeck measurements. The theory models the increase in the tilt angle up to 85° degrees, as shown in Figure S14.

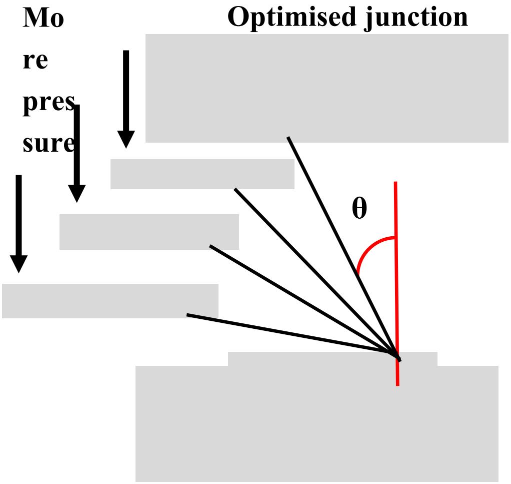

Figure S15. Schematic illustrations the modeling of increasing the tilt angle (pressure model).

2.7 DFT Calculations

In the following transport calculations, the ground state Hamiltonian and optimized geometry of each compound was obtained using the density functional theory (DFT) code.[17] The local density approximation (LDA) exchange correlation functional was used along with double zeta polarized (DZP) basis sets and the norm conserving pseudo potentials. The real space grid was defined by a plane wave cut-off of 250 Ry. The geometry optimization was carried out to a force tolerance of 0.01 eV/Å. This process was repeated for a unit cell with the molecule between gold electrodes where the optimized distance between Au and the thiol anchor group was found to be 2.4 Å. From the ground state Hamiltonian, the transmission coefficient, the room temperature electrical conductance G and Seebeck coefficient S was obtained, as described in the sections below. We model the properties of a single molecule in the junction as previous works[18] have shown that the calculated conductance of a SAM differs only slightly from that of single molecules.

2.8 Transport Calculations

The transmission coefficient curves *T(E),* obtained from using the Gollum transport code, were calculated for molecules **1** and **2** based on the pressure model (tilt angle). The HOMO resonance is predicted to be pinned near the Fermi Level of the electrodes for the two molecules, however, we set the Fermi Level to be in the mid gap at approximately 0.5 eV (black-dashed line), as shown in Figures S15 and S16.

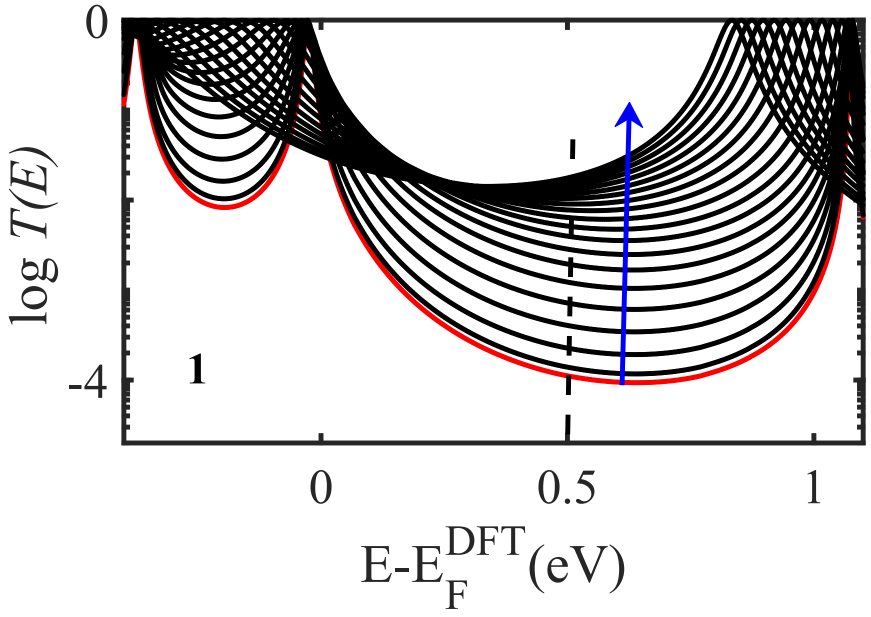

Figure S16. Zero bias transmission coefficient *T(E)* of molecule **1** as a function of pressure. The tilt angle varies from approximately 55° (red curve) to 80°, (red-line light pressure and blue arrow points to toward heavy pressure, for clarity not all curves are shown).

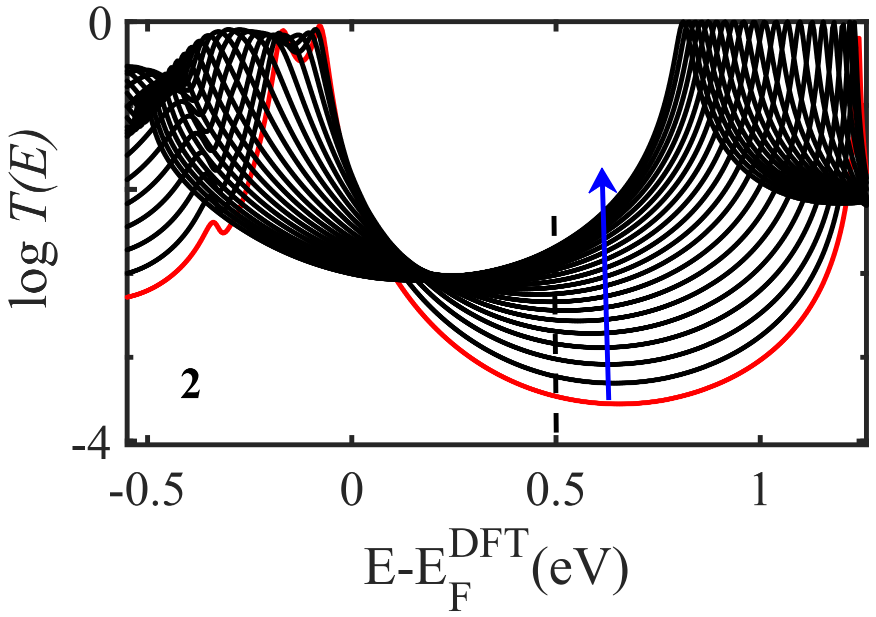

Figure S17. Zero bias transmission coefficient *T(E)* of molecule **2** as a function of pressure. The tilt angle varies from approximately 55° (red curve) to 80°, (red-line light pressure and blue arrow points to toward heavy pressure, for clarity not all curves are shown).

2.9 Seebeck coefficient

After computing the electronic transmission coefficients for the two molecules, thermoelectric properties such as their Seebeck coefficient S were computed.

To calculate the Seebeck cofficient of these molecular junctions, it is useful to introduce the non-normalised probability distribution $P(E)$ defined by

$$P(E) = -T(E)\frac{df(E)}{dE} \tag{S2}$$

where $f(E)$ is the Fermi-Dirac function and $T(E)$ are the transmission coefficients and whose moments L_i are denoted as follows

$$L_i = \int dEP(E)(E - E_F)^i \tag{S3}$$

where E_F is the Fermi energy. The Seebeck cofficient S, is then given by

$$S(T) = -\frac{1}{|e|T}\frac{L_1}{L_0} \qquad \text{(S4)}$$

where e is the electronic charge.

Supplementary Figures S17 and S18 shows the thermopower S evaluated at room temperature for different energy ranges $E_F - E_F^{DFT}$ as a function of pressure.

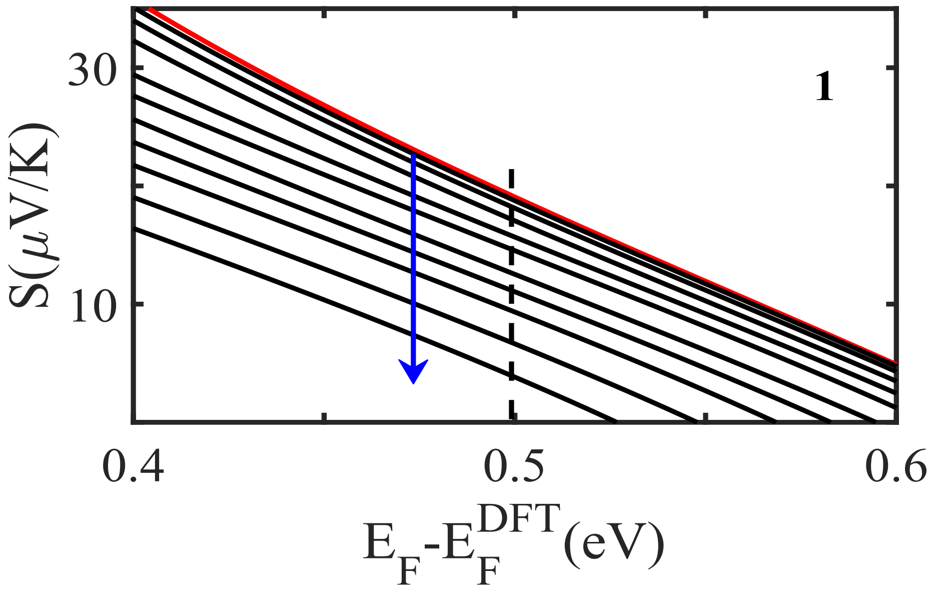

Figure S18. Seebeck coefficient S of molecule **1** as a function of pressure. The tilt angle varies from approximately 55º (red curve) to 80º, (red-line light pressure and blue arrow points to toward heavy pressure, for clarity not all curves are shown).

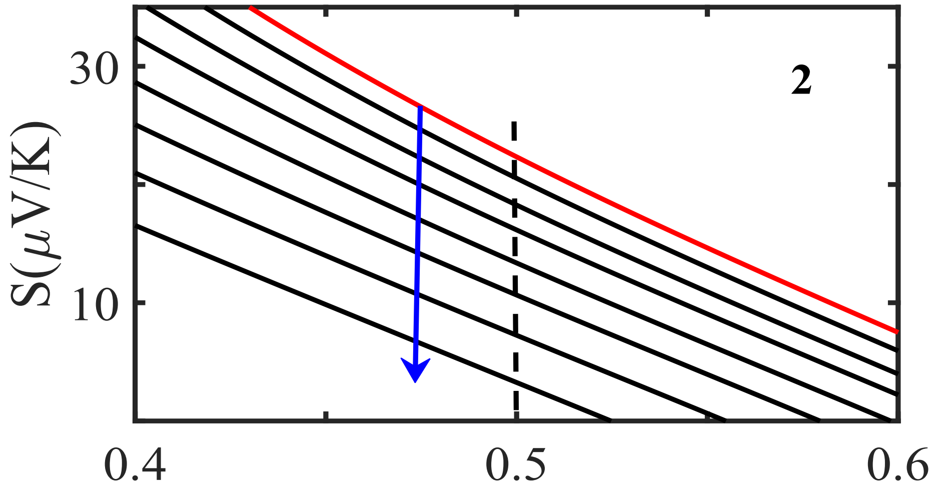

Figure S19. Seebeck coefficient S of molecule **2** as a function of pressure. The tilt angle varies from approximately 55° (red curve) to 80°, (red-line light pressure and blue arrow points to toward heavy pressure, for clarity not all curves are shown).

2.10 Mechanical gating charge transport in molecular junctions

In this section, the $I - V$ curves were calculated for each tilt angle for both SAMs **1** and **2** as shown in Figure S19**.** The next step is to calculate I/V for **1** and **2** as show in Figure S20. Finally, Figure S21 shows a two-dimensional of I/V plotted versus the bias voltage for both **1** and **2**.

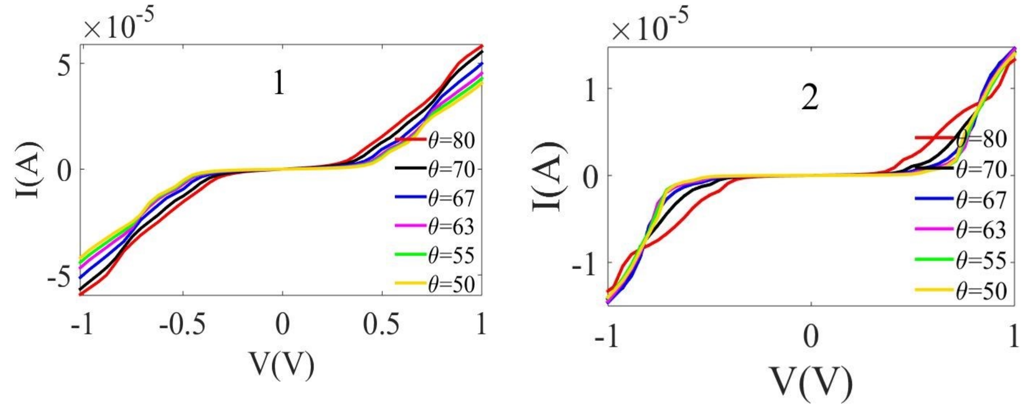

Figure S20. Current transport in molecular junctions. Current plotted versus bias voltage for SAMs **1** and **2** (left and right respectively).

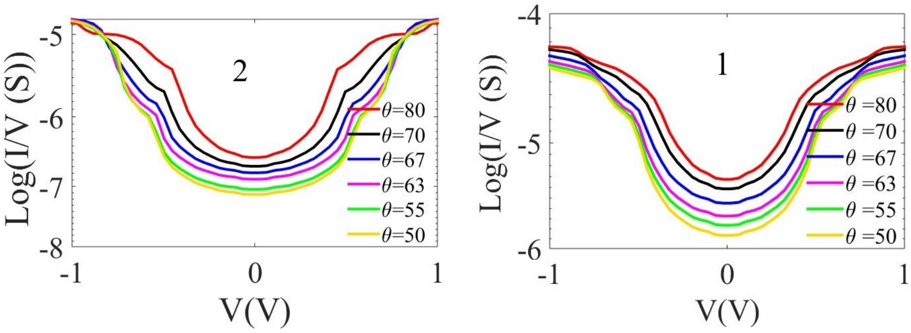

Figure S21. I/V plotted versus bias voltage for SAMs **1** and **2** (left and right respectively).

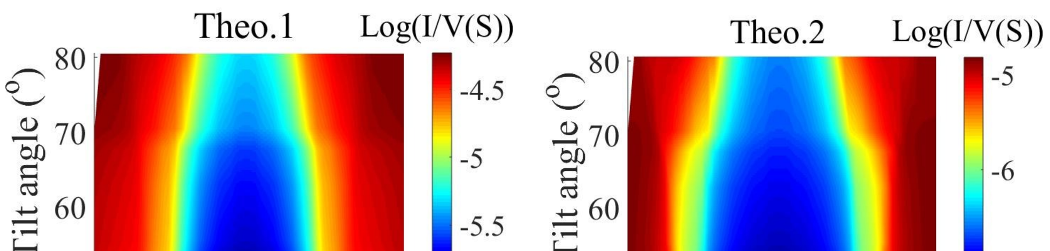

Figure S22. Two-dimensional visualization of dI/dV plotted versus bias voltage for SAMs **1** and **2**.

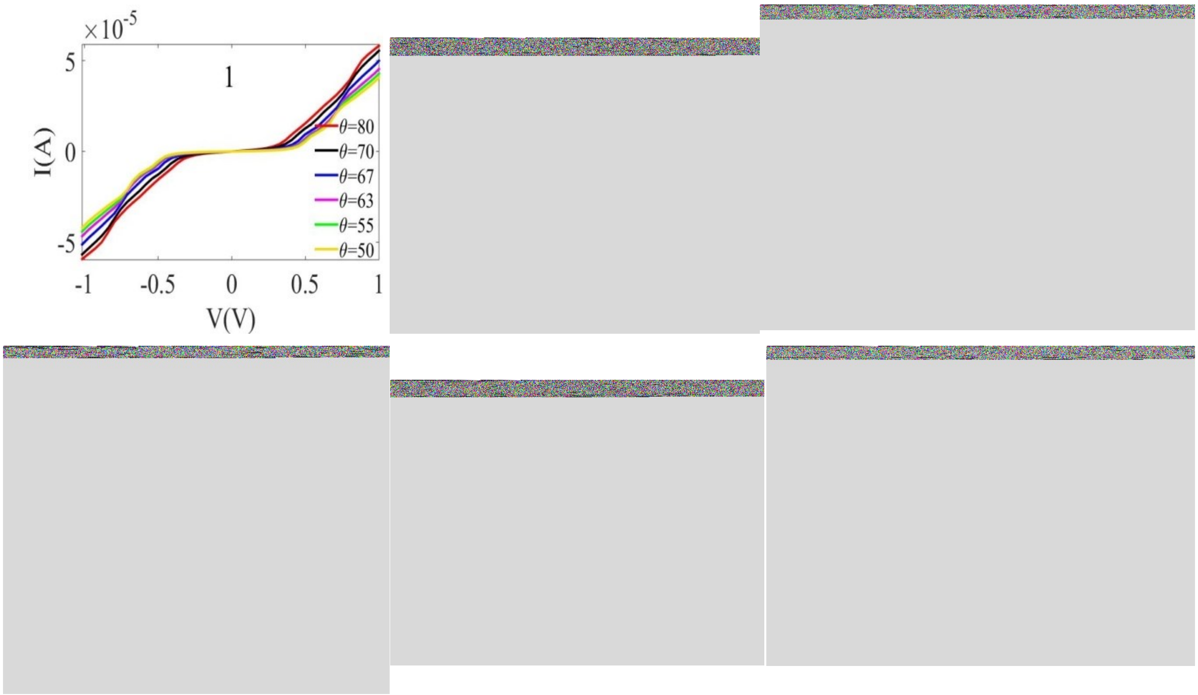

Figure S23. $I-V$ curves, I/V curves and two-dimensional visualization of I/V plotted versus bias voltage for SAMs **1** and **2**.

3. References

1. Weiss, E. A.; Kaufman, G. K.; Kriebel, J. K.; Li, Z.; Schalek, R.; Whitesides, G. M., Si/SiO2-Templated formation of ultraflat metal surfaces on glass, polymer, and solder supports: Their use as substrates for self-assembled monolayers. *Langmuir* **2007,** *23* (19), 9686-9694.
2. Banner, L. T.; Richter, A.; Pinkhassik, E., Pinhole-free large-grained atomically smooth Au(111) substrates prepared by flame-annealed template stripping. *Surf Interface Anal* **2009,** *41* (1), 49-55.
3. Sauerbrey, G., Verwendung Von Schwingquarzen Zur Wagung Dunner Schichten Und Zur Mikrowagung. *Z Phys* **1959,** *155* (2), 206-222.

4. Rospigliosi, A.; Ehlich, R.; Hoerber, H.; Middelberg, A.; Moggridge, G., Electron transfer of plurimodified DNA SAMs. *Langmuir* **2007,** *23* (15), 8264-8271.
5. Yakuphanoglu, F.; Okur, S.; Ozgener, H., Modification of metal/semiconductor junctions by self-assembled monolayer organic films. *Microelectron Eng* **2009,** *86* (11), 2358-2363.
6. Wang, X.; Bennett, T. L.; Ismael, A.; Wilkinson, L. A.; Hamill, J.; White, A. J.; Grace, I. M.; Kolosov, O. V.; Albrecht, T.; Robinson, B. J., Scale-up of room-temperature constructive quantum interference from single molecules to self-assembled molecular-electronic films. *Journal of the American Chemical Society* **2020,** *142* (19), 8555-8560.
7. Song, H.; Lee, H.; Lee, T., Intermolecular chain-to-chain tunneling in metal-alkanethiol-metal junctions. *J Am Chem Soc* **2007,** *129* (13), 3806-+.
8. Dokukin, M. E.; Sokolov, I., Quantitative mapping of the elastic modulus of soft materials with HarmoniX and PeakForce QNM AFM modes. *Langmuir* **2012,** *28* (46), 16060-16071.
9. Young, T.; Monclus, M.; Burnett, T.; Broughton, W.; Ogin, S.; Smith, P., The use of the PeakForceTM quantitative nanomechanical mapping AFM-based method for high-resolution Young's modulus measurement of polymers. *Measurement Science and Technology* **2011,** *22* (12), 125703.
10. Wang, G.; Kim, T. W.; Jo, G.; Lee, T., Enhancement of Field Emission Transport by Molecular Tilt Configuration in Metal-Molecule-Metal Junctions. *J Am Chem Soc* **2009,** *131* (16), 5980-5985.
11. Soler, J. M.; Artacho, E.; Gale, J. D.; García, A.; Junquera, J.; Ordejón, P.; Sánchez-Portal, D., The SIESTA method for ab initio order-N materials simulation. *Journal of Physics: Condensed Matter* **2002,** *14* (11), 2745.
12. Artacho, E.; Anglada, E.; Diéguez, O.; Gale, J. D.; García, A.; Junquera, J.; Martin, R. M.; Ordejón, P.; Pruneda, J. M.; Sánchez-Portal, D. J. J. o. P. C. M., The SIESTA method; developments and applicability. **2008,** *20* (6), 064208.
13. Ismael, A. K.; Lambert, C. J. J. J. o. M. C. C., Single-molecule conductance oscillations in alkane rings. **2019,** *7* (22), 6578-6581.
14. Gantenbein, M.; Wang, L.; Al-jobory, A. A.; Ismael, A. K.; Lambert, C. J.; Hong, W.; Bryce, M. R. J. S. r., Quantum interference and heteroaromaticity of para-and meta-linked bridged biphenyl units in single molecular conductance measurements. **2017,** *7* (1), 1-9.
15. Ismael, A.; Wang, X.; Bennett, T. L.; Wilkinson, L. A.; Robinson, B. J.; Long, N. J.; Cohen, L. F.; Lambert, C. J. J. C. S., Tuning the thermoelectrical properties of anthracene-based self-assembled monolayers. **2020,** *11* (26), 6836-6841.
16. Ismael, A.; Al-Jobory, A.; Wang, X.; Alshehab, A.; Almutlg, A.; Alshammari, M.; Grace, I.; Benett, T. L.; Wilkinson, L. A.; Robinson, B. J. J. N. A., Molecular-scale thermoelectricity: as simple as 'ABC'. **2020**.
17. Soler, J. M.; Artacho, E.; Gale, J. D.; García, A.; Junquera, J.; Ordejón, P.; Sánchez-Portal, P., The SIESTA method for ab initio order- N materials simulation. *J. Phys.: Condens. Matter* **2002,** *14* (11), 2745.

18. Herrer, L.; Ismael, A.; Martin, S.; Milan, D. C.; Serrano, J. L.; Nichols, R. J.; Lambert, C.; Cea, P., Single molecule vs. large area design of molecular electronic devices incorporating an efficient 2-aminepyridine double anchoring group. *Nanoscale* **2019,** *11* (34), 15871-15880.
